# Predictors of Radiotherapy Induced Bone Injury (RIBI) after stereotactic lung radiotherapy

**DOI:** 10.1186/1748-717X-7-159

**Published:** 2012-09-17

**Authors:** Mojgan Taremi, Andrew Hope, Patricia Lindsay, Max Dahele, Sharon Fung, Thomas G Purdie, David Jaffray, Laura Dawson, Andrea Bezjak

**Affiliations:** 1 Radiation Medicine Program, Princess Margaret Hospital, Toronto, ON, Canada; 2 Department of Radiation Oncology, University of Toronto, Toronto, ON, Canada; 3 Department of Biostatistics, Princess Margaret Hospital, Toronto, ON, Canada; 4 Department of Radiation Oncology, VU University Medical Center, Amsterdam, the Netherlands; 5 Radiation Oncology Department, Stronach Cancer Center, Newmarket, ON, Canada

**Keywords:** Stereotactic body radiotherapy, Radiotherapy toxicity, Rib fracture, Nomogram, Non-small cell lung cancer, Chest wall pain

## Abstract

**Background:**

The purpose of this study was to identify clinical and dosimetric factors associated with radiotherapy induced bone injury (RIBI) following stereotactic lung radiotherapy.

**Methods:**

Inoperable patients with early stage non-small cell lung cancer, treated with SBRT, who received 54 or 60 Gy in 3 fractions, and had a minimum of 6 months follow up were reviewed. Archived treatment plans were retrieved, ribs delineated individually and treatment plans re-computed using heterogeneity correction. Clinical and dosimetric factors were evaluated for their association with rib fracture using logistic regression analysis; a dose-event curve and nomogram were created.

**Results:**

46 consecutive patients treated between Oct 2004 and Dec 2008 with median follow-up 25 months (m) (range 6 – 51 m) were eligible. 41 fractured ribs were detected in 17 patients; median time to fracture was 21 m (range 7 – 40 m). The mean maximum point dose in non-fractured ribs (n = 1054) was 10.5 Gy ± 10.2 Gy, this was higher in fractured ribs (n = 41) 48.5 Gy ± 24.3 Gy (p < 0.0001). On univariate analysis, age, dose to 0.5 cc of the ribs (D_0.5_), and the volume of the rib receiving at least 25 Gy (V_25_), were significantly associated with RIBI. As D_0.5_ and V_25_ were cross-correlated (Spearman correlation coefficient: 0.57, p < 0.001), we selected D_0.5_ as a representative dose parameter. On multivariate analysis, age (odds ratio: 1.121, 95% CI: 1.04 – 1.21, p = 0.003), female gender (odds ratio: 4.43, 95% CI: 1.68 – 11.68, p = 0.003), and rib D_0.5_ (odds ratio: 1.0009, 95% CI: 1.0007 – 1.001, p < 0.0001) were significantly associated with rib fracture.

Using D_0.5,_ a dose-event curve was constructed estimating risk of fracture from dose at the median follow up of 25 months after treatment. In our cohort, a 50% risk of rib fracture was associated with a D_0.5_ of 60 Gy.

**Conclusions:**

Dosimetric and clinical factors contribute to risk of RIBI and both should be included when modeling risk of toxicity. A nomogram is presented using D_0.5_, age, and female gender to estimate risk of RIBI following SBRT. This requires validation.

## Background

SBRT has superior local tumor control when compared to conventionally fractionated radiotherapy [1]. However due to the large doses per fraction, the risk of late normal tissue toxicities such as radiation induced bone injury (RIBI) such as rib fracture may be increased [2]. Rib fracture following SBRT has been reported by a number of groups [3-5] including our own [5] - we previously found that out of 42 patients treated with 54 or 60 Gy in 3 fractions, 9 patients developed a total of 15 fractured ribs after a median follow-up of 17 months. The median radiation dose to the fractured rib was 50.1 Gy. The current report explores in detail the relationship of rib dose to subsequent rib fractures risk in a larger group with longer follow up. The primary objective of this study was to identify dosimetric and clinical risk factors for RIBI. The secondary objective was to generate a nomogram estimating risk of rib fracture from these factors.

## Methods

From Oct 2004 to Dec 2008, 127 medically inoperable patients with T1-2N0M0 non-small cell lung carcinoma (NSCLC) were treated on a prospective institutional research ethics board-approved lung SBRT protocol at Princess Margaret Hospital. Written consent was obtained to participate on study and to collect the data for publication. Patients were treated with several dose fractionation schedules: 5 Gy × 10 fractions (n = 12), or 7.5 Gy × 8 fr (n = 10) for centrally located tumors, and for peripheral tumors 12 Gy × 4 fr (n = 52), 18 Gy or 20 Gy (the latter was used prior to heterogeneity correction) × 3 fr (n = 53) [6] . Ribs or chest wall were not explicitly considered a critical structure at the time of these patients’ treatment planning. Post-treatment, the follow up schedule included clinic visits and thoracic imaging - chest x-ray 6 weeks after SBRT and chest CT scan at 3, 6, 9 and 12 months, every 6 months in the second year and yearly thereafter. The Common Terminology Criteria for Adverse Events (CTCAE) v3.0 was used to score acute and late toxicity [7]. A subset of consecutive patients treated with 18 or 20 Gy × 3 fractions and with more than 6 months follow up was selected for this study as we had previously observed rib fractures in this group and we had not observed fractured ribs with other schedules such as 48 Gy in 4 fractions or 50 Gy in 10 fractions.

### Detecting fractured ribs

Because the radiology reports inconsistently reported fractured ribs and some rib fractures are known to be asymptomatic [5], identification of RIBI was systematically performed in three steps: 1) abstracting information from serial imaging reports, 2) review of all serial follow up imaging by two independent observers (a radiation oncology fellow and a radiology fellow). Any cases with discrepancy were discussed to obtain agreement, 3) 20% of all RIBI events were reviewed randomly by a staff radiologist resulting in 100% agreement on the fracture site and 88% agreement on the fracture date (defined as the date that the first sign of periosteal distortion was observed). In the cases with date discrepancy, the radiologist detected the fractured ribs on the scan performed 6 months earlier. Grading of rib fractures was performed using the radiological as well as clinical prospectively collected toxicity data, as per CTCAE v3.0 [7], rib fractures were graded radiologically and clinically from prospective toxicity data (Table 1).

**Table 1:** Common Toxicity Criteria for Adverse Events v3.0 (CTCAE) for fracture and pain

**Adverse event**	**Grade 1**	**Grade2**	**Grade3**	**Grade4**	**Grade5**
Fracture	Asymptomatic, Radiologic findings only	Symptomatic but non-displaced	Symptomatic and displaced or open wound with bone exposure	Disabling	
Pain	Mild pain not interfering with function	Moderate pain, Pain or analgesics interfering with function but not interfering with ADL	Sever pain, pain or analgesics	Disabling	-
			severely interfering with ADL		

### Dosimetric evaluation

The majority of the patients had a respiratory-correlated CT scan (4D CT) for their treatment planning (32/46 patients, 70%). In the remaining 14 patients (treated early in our SBRT program), 4D CT was used to assess tumor motion, but not for treatment planning. Typically the primary data set used for treatment planning was the maximum exhale phase of the 4D CT, and less often a helical CT data set.

To obtain dosimetric rib data, each rib was individually contoured on the primary CT data set used for SBRT treatment planning. Ribs were delineated from the costovertebral to the costosternal/costocartilage area bilaterally, using threshold contouring tools (1080 to 2400 HU) and with manual review and correction in the radiation treatment planning system (Pinnacle, v8.0, Philips Medical Systems, Fitchburg, WI, USA). A representative diagnostic CT scan showing the fractured rib(s) for each patient with RIBI was registered to the treatment planning CT scan using the fractured rib as the region of interest for image fusion. The fracture site was contoured by a single observer (MT) and 3D CT registration information (x, y and z) for each fractured rib and callus were documented for quality assurance (QA) purposes. A staff radiation oncologist reviewed and approved a subset of the contoured fractures with high levels of agreement. It is important to note that although the analysis was performed using the maximum point dose to the ribs, in 35 fractures (14 patients) this was not the same as the maximum dose to the fracture site. The most likely explanation was considered to be contouring subjectivity and difficulty in determining the exact fracture site boundaries*.*

The dose calculation grid (resolution of 0.25 cm × 0.25 cm × 0.25 cm) was adjusted in all patients to cover all ribs and each SBRT plan was re-computed with heterogeneity correction [8] while maintaining the planned monitor units.

Resulting planning data was exported using the RTOG format and the dosimetric information extracted using CERR (Computational Environment for Radiotherapy Research) [9].

### Data collection and analysis

Clinical patient data was extracted from the prospectively collected institutional SBRT database. This included: age, sex, comorbidities (chronic obstructive pulmonary disease (COPD), diabetes mellitus (DM)), number/location/date of fractured ribs, history of traumatic rib fractures, tumor size, date of SBRT treatment, date of last follow up or death and a history of cancer metastasis to the bone.

Dosimetric data extracted from the re-computed plans and dose volume histogram (DVH) included: rib D_V_ (minimum absolute dose received by volume V), ribs V_D_ (absolute volume receiving at least dose D), maximum/mean/median point dose to the ribs, GTV (gross tumor volume), the minimum 3D distance between the GTV and any rib, the minimum 3D distance between the GTV and any fractured ribs, and cumulative dose-volume histogram (DVH) for each individual rib.

The correlation between dose and volume was examined using the Spearman correlation. Univariate logistic regression was used to test the association of various predictors with the risk of fracture. Since each patient could have multiple fractures, repeated measures have been taken into consideration.

A modified stepwise model fitting process was used to select the best fit multivariate model. Maximum likelihood estimation was used to select thresholds for dose and volume. All analyses were performed using SAS v9.1 for Windows TM and all reported p-values were 2-sided, a p-value of < 0.05 was considered significant. Using the multivariate model, a nomogram was generated and its receiver operating characteristic (ROC) calculated to assess its discrimination power.

A final logistic model was generated estimating RIBI risk at a median follow up of 25 month based on the ‘all rib’ analysis.

Probability of fracture:

$$P=\frac{1}{1+e^{-\left(a+hX\right)}}$$

P is the probability of a fracture (1), *e* is the base of the natural logarithm (about 2.7); *a* and *b* are the parameters of the model. The value of *a* yields P when X is zero, and *b* adjusts how quickly the probability changes with changing X.

## Results

### Patient characteristics

From Oct 2004 to Dec 2008, 48 consecutive patients were treated with 18 or 20 Gy × 3 fractions and followed for > 6 months, two were excluded from this analysis - one had rib fracture at baseline, pre-SBRT, the other had rib fracture associated with a bone metastasis. Thus, 46 patients with 49 tumors (3 patients had 2 tumors) were analyzed. Median age was 73 years (range: 48 to 89 years) and median follow up was 25 months (range: 6 to 51 m). There were 22 male and 24 female patients with similar median age (73 year) but median follow-up was slightly higher in female group (26.2 vs. 22.7 months) as shown in Table 2. 17 of 46 patients (37%) were identified as having developed rib fractures with a total of 41 fractured ribs and 43 fracture sites. Of 17 patients with fractured ribs, 11 (with 30 fractures) were female and 6 (with 13 fractures) were male (Table 2).

**Table 2:** Clinical factors in 46 patients treated with lung SBRT

	**Total**	**Female**	**Male**
Patients	46	24	22
Median Age (year)	72.8	72.6	72.8
(range)	(48.3-89.6)	(58-89.6)	(48.3-85.5)
Median follow up time (Months)	24.9	26.2	22.7
(range)	(6-51.2)	(6-51.2)	(7.6-48.5)
Number of patients with rib fracture	17	11	6
Number of fractured sites	43	30	13
8 pts with DM* Patients with no fracture	6	1	5
Patients with fracture	2	0	2
29 pts COPD** Patients with no fracture	18	9	9
Patients with fracture	11	7	4
Mean (± SD) Tumor size (cm)	2.6 ± 1.2	2.7 ± 1.2	2.6 ± 1.2
Closest 3 dimensional distance from tumor to the ribs (cm)	0.96	1.01	0.88
(range)	(0 – 3.28)	(0 – 3.28)	(0 – 2.76)

*DM: Diabetes Mellitus, ** COPD: Chronic Obstructive Pulmonary Disease.

**Figure 1:**
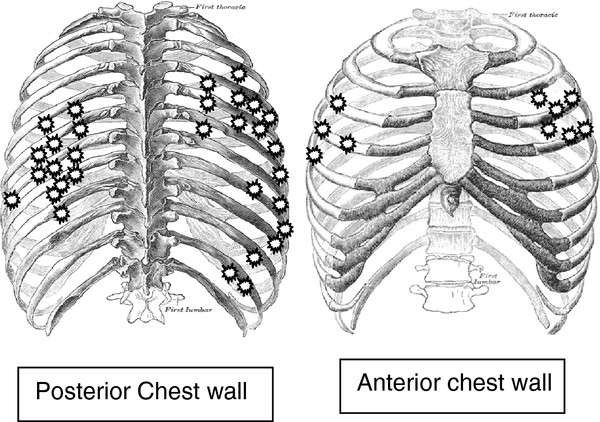
Anatomic locations of 41 fractured ribs in 17 patients with RIBI.

Anatomic locations of fractured ribs are shown in Figure 1. In patients with multiple rib fractures, the fracture sites were in proximity to each other (Table 3). Two patients had bilateral fractured ribs however the dose to the fractured ribs was so low in one of these patients (pt # 9 in table 3) that radiotherapy cannot be considered the primary risk factor. In such cases other clinical factors may play the more important role.

**Table 3:** Max point dose to the callus in 17 patients with rib fractures (43 calluses in 41 fractured ribs)

**Patients N = 17**	**Number of rib fractures N = 41**	**Callus N = 43**	**Callus max point dose (Gy)**	**Highest max Point dose to fractured rib (Gy)**	**Highest max point dose to callus (Gy)**	**Lowest max point dose to fractured rib (Gy)**	*Mean dose (Gy)
1	2			68.52 *Lt rib 5*	68.52 *Lt rib 5*	61.85 *Lt rib 6*	65.18
		***Lt rib 5***	***68.52***				
		***Lt rib 6***	***62.40***				
2	6			76.39 *Rt rib 5*	73.6 *Rt rib 5*	6.80 *Rt rib 11*	41.59
		***Rt rib 4***	***36.27***				
		***Rt rib 5***	***73.6***				
		***Rt rib 6***	***29.45***				
		***Rt rib 9***	***6.06***				
		***Rt rib 10***	***7.58***				
		***Rt rib 11***	***1.16***				
3	2			64.63 *Rt rib 4*	61.54 *Rt rib 4*	23.15 *Rt rib 3*	43.89
		***Rt rib 3***	***23.15***				
		***Rt rib 4***	***61.454***				
4	4			88.05 *Rt rib 6*	87.91 *Rt rib 6*	13.17 *Rt rib 4*	50.61
		***Rt rib 3***	***24.07***				
		***Rt rib 4***	***13.17***				
		***Rt rib 5***	***68.39***				
		***Rt rib 6***	***87.91***				
5	1	***Rt rib 4***	***48.54***	50.10	48.54	48.54	49.32
6	2			59.56 *Rt rib 5*	29.76 *Rt rib 5*	25.03 *Rt rib 4*	42.29
		***Rt rib 4***	***25.03***				
		***Rt rib 5***	***29.76***				
7	2			69.36 *Rt rib 4*	58.79 *Rt rib 3*	49.05 *Rt rib 4*	59.20
		***Rt rib 3***	***58.79***				
		***Rt rib 4***	***49.5***				
8	1	***Rt rib 5***	***35.12***	35.84	35.12	35.12	35.48
9	3			21.82 *Rt rib 7*	0.7 *Rt rib 7*	0.45 *Rt rib 8*	11.26
		***Lt rib 7***	***0.48***				
		***Rt rib 7***	***0.7***				
		***Rt rib 8***	***0.45***				
10	2			71.39 *Rt rib 3*	70.84 *Rt rib 3*	23.37 *Rt rib 2*	47.38
		***Rt rib 2***	***23.37***				
		***Rt rib 3***	***70.84***				
11	4			75.34 *Lt rib 6*	72.59 *Lt rib 6*	6.13 *Lt rib 8*	40.73
		***Lt rib 5***	***68.39***				
		***Lt rib 6***	***72.59***				
		***Lt rib 7***	***48.85***				
		***Lt rib 8***	***3.25***				
12	3			69.86 *Rt rib 4*	69.86 *Rt rib 4*	10.64 *Rt rib 5*	40.25
		***Rt rib 4***	***69.86***				
		***Rt rib 5***	***10.64***				
		***Rt rib 5***	***68.37***				
		***Rt rib 6***	***32.04***				
13	2			68.49 *Lt rib 7*	66.40 *Lt rib 7*	12.16 *Lt rib 6*	40.32
		***Lt rib 6***	***62.03***				
		***Lt rib6***	***12.16***				
		***Lt rib 7***	***66.40***				
14	2			50.38 *Lt rib 9*	50.38 *Lt rib 9*	44.04 *Lt rib 8*	47.21
		***Lt rib 8***	***44.04***				
		***Lt rib 9***	***50.38***				
15	3			72.44 *Lt rib 7*	69.07 *Lt rib 7*	23.46 *Rt rib 5*	47.95
		***Rt rib 5***	***23.46***				
		***Lt rib 7***	***69.07***				
		***Lt rib 8***	***66.96***				
16	1	***Rt rib 11***	***0.10***	0.56	0.1	0.10	0.33
17	1	***Rt rib 5***	***44.07***	64.18	44.07	44.07	54.12

Max point dose to the fractured rib was not located on the callus in 14/17 patients.

* Mean dose is the average of the lowest and highest maximum point doses to the fractured rib(s).

**Figure 2:**
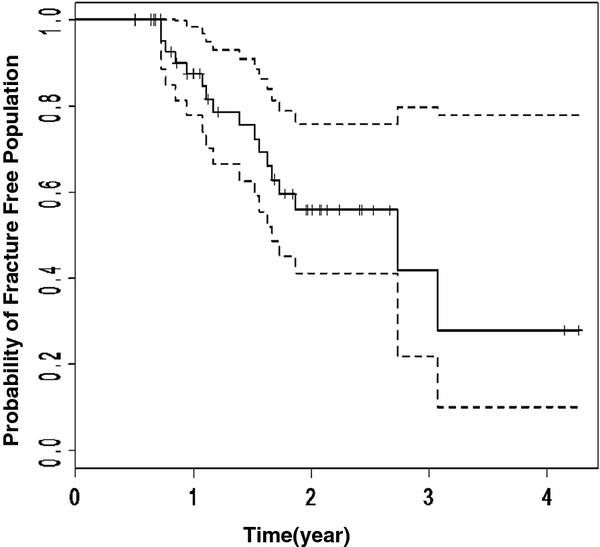
**Kaplan Meier curve for fractured rib as an event (n = 46 patients). **Dashed lines indicate 95% confidence intervals.

Median time to development of a fractured rib was 21 months (range: 7 - 40 m) as shown in Figure 2. Thirteen of 17 patients with rib fracture had at least two fractured sites. Detailed dosimetric information for each fractured rib and the callus in 17 patients with rib fracture has been summarized in table 3. Of patients identified with fractures, the original radiologic reports did not report fracture in 3 out of 17 patients (18%). In those patients in whom rib fractures were reported, the number and first reported date of fracture were incomplete. Overall, a total of 15 out of 41 rib fractures (37%) were not noted in the original report and the first date of reported fracture was on average 5 months (range: 0 to 18 m) later than was detected in this study.

**Figure 3:**
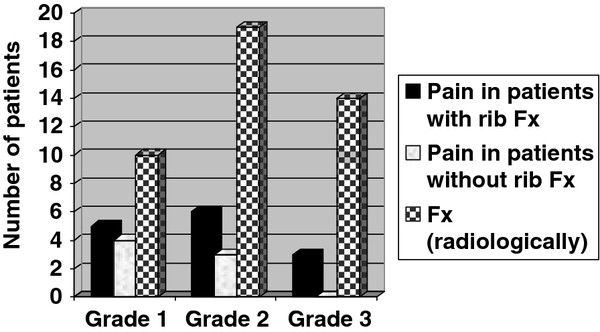
Grading of chest wall pain (n = 21 patients with reports of chest wall pain > 0) and rib fractures (n = 17 patients, 43 fractures) based on CTCAE criteria.

Clinical (chest wall pain) and radiologic (rib fracture) toxicities are shown in Figure 3. Chest wall pain was detected in 7/29 patients (24%) without rib fracture and in 14/17 patients (82%) with rib fractures. Although in the majority of patients fractured ribs remain unhealed, patients did not require narcotic pain medications for a long time. In all patients except one (with 6 fractured ribs), pain became more stable after 6–8 months.

Patients with chest wall pain received higher dose of radiation to the ribs compared to patients without chest wall pain (62.76 Gy, range: 28.4-88.05 Gy vs. 47.21 Gy, range: 15.9-73.19 Gy; p value: 0.008) (Table 4).

**Table 4:** Mean maximum point dose to the ribs in patients with or without chest wall pain

**Group**	**Number of patients**	**Mean maximum point dose (Gy)**	**p-value**
		**(range)**	
**Patients with chest wall pain**	21	62.76	0.008*
		(28.4 - 88.05)	
**Patients without chest wall pain**	25	47.21	
		(15.9 - 73.19)	

*Wilcoxon-Mann-Whitney test was used to obtained the p-value.

### Dosimetric factors

After re-contouring, 1095 ribs were available for analysis; in some patients some of the whole ribs could not be contoured because they were not fully included in the planning CT scan images (less than 5% in ribs 1 and 2 but more than 50% in ribs 11 and 12).

All individual fracture sites were contoured separately however in the majority of cases (35 fracture sites in 14 patients) the maximum dose to the fracture site was not the maximum dose to the fractured rib therefore as mentioned above the analysis was performed using the maximum point dose to the ribs.

Analysing per patient, using the maximum dose received by any rib in each patient, a significant difference (p = 0.02) was noted between 29 patients with no rib fracture (50.2 Gy ± 17.7 Gy, range: 21.6 to 73.2 Gy) vs. 17 patients with rib fracture (63.7 Gy ± 15.3 Gy, range: 26.6 to 88 Gy). There was no significant difference (p = 0.09) between the mean maximum dose to the first fractured rib (52 Gy +/−24.9 Gy, range: 3.9 - 76.4 Gy) compared to subsequent fractured ribs (50 Gy+/− 19 Gy, range: 19.6 - 71.2 Gy).

Assuming each rib was independent, out of 1095 ribs, 41 had fractures and 1054 did not. In non-fractured ribs, the mean maximum point dose was 10.5 Gy ± 10.2 Gy (range: 0.2 to 87 Gy) compared to 48.5 Gy ± 24.3 Gy (range 0.6 to 88 Gy) in fractured ribs; this was statistical significantly different (p < 0.001).

**Figure 4:**
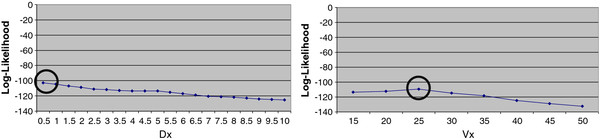
**Maximum likelihood curve for fractured ribs. **Dx: Absolute dose to a certain volume (0.5-10 cc) of the ribs. Vx: Absolute volume receiving certain dose (15-50 Gy) of the ribs.

While many dosimetric parameters were correlated with rib fracture, D_0.5_ and V_25_ appeared to have the highest individual correlations (Figure 4).

**Figure 5:**
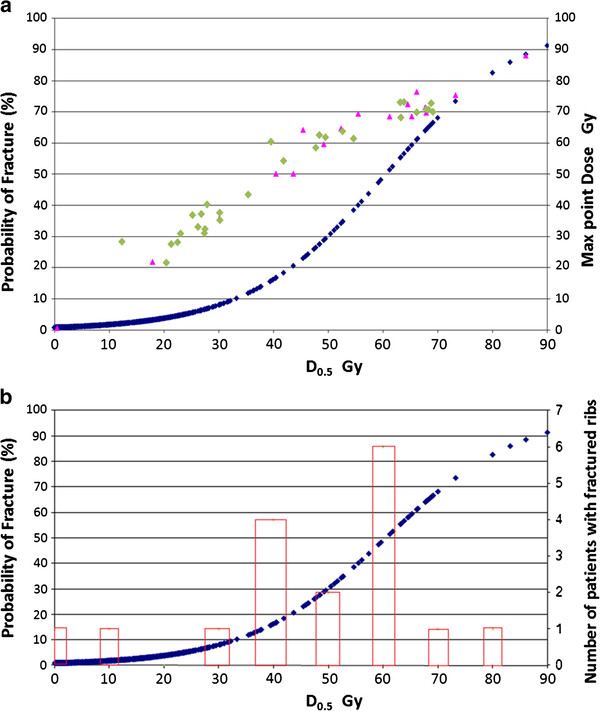
**a: D**_**0.5 **_**for patients with fractured ribs (pink triangle) and without fractured ribs (Green diamond); calculated probability of fracture (blue diamond) at the median follow up of 25 months based on D**_**0.5 **_**. b: Distribution of 17 patients with fractured rib per D**_**0.5 **_**dose groups (10 Gy bin size), and calculated probability of fracture (blue diamond).**

To evaluate the impact of including ribs receiving very low dose of radiotherapy on correlations, ribs receiving less than 1, 5, 10, 15, 20 and 25 Gy were sequentially excluded from the profile-likelihood modeling process. Both D_0.5_ and V_25_ were well correlated in all sub-groups. As D_0.5_ and V_25_ were cross-correlated (Spearman correlation coefficient: 0.57, p < 0.001), we selected D_0.5_ as a representative dose parameter that could be included in subsequent modeling efforts. Using D_0.5,_ a dose-event curve was constructed estimating risk of fracture from dose at the median follow up of 25 months after treatment (Figure 5).

### Combining clinical and dosimetric factors

On univariate analysis, correlations with RIBI were found with age (p = 0.045), but not with gender, COPD or diabetes. In terms of dosimetric factors, all D_x_ and V_x_ were significant on univariate analysis, as discussed above; D_0.5_ was used for multivariate analysis.

On multivariate analysis, age (p = 0.003), female gender (p = 0.003) and rib D_0.5_ (p < 0.0001) were variables that were significantly associated with RIBI (Table 5).

**Table 5:** Univariate and multivariate analysis on predictors for rib fractures (repeated measures have been taken into consideration)

**Univariate analysis**			
**Predictor**	**Odds Ratio**	**95% CI**	**p-value**
Age (years)	1.083	1.002 - 1.172	0.045
Gender-F	2.256	0.656 - 7.756	0.2
Diabetes Mellitus-yes	0.51	0.091 - 2.876	0.45
COPD-yes	0.97	0.275 – 3.386	0.96
Tumor size	1.037	0.982 -1.095	0.19
Smallest 3D distance between the tumor and closest rib	0.408	0.152 – 10.970	0.07
**Multivariate analysis**			
Age (year)	1.121	1.04 – 1.21	0.003
Gender-F	4.43	1.68 – 11.68	0.003
D_0.5_	1.0009	1.0007 - 1.0011	<0.0001

A nomogram was generated based on this multivariate model. The nomogram estimates risk of RIBI at 25 months median follow up in our cohort of patients (Figure 4) based on pre-treatment factors including age, gender and D_0.5_ in patients treated with 54 or 60 Gy in 3 fractions.

**Figure 6:**
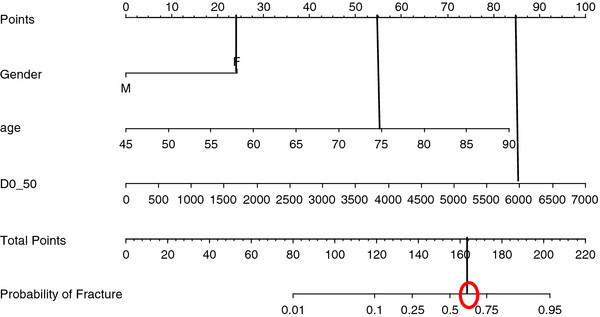
**RIBI nomogram based on gender, age and D**_**0.5 **_**in 46 patients treated with SBRT at Princess Margaret Hospital (Estimating risk of rib fracture at median follow up of 25 month). **Risk of rib fracture in a 75 year old lady treated with 54 Gy in 3 fractions and D0.5 of 60 Gy (within a median FU of 2 years)is about 65%.

Although, the nomogram still needs validation, it may be helpful in estimating the risk of rib fracture in an individual patient. For example estimated risk of rib fracture in a 75 year old (55 points for age) lady (25 points for female gender) with D_0.5_ of 60 Gy (85 points for dose) is about 65% (total of 165 points as shown in Figure 6), which is much higher than in a same age man with the same planning criteria (risk of rib fracture of 15-20%). This emphasizes the importance of clinical factors when estimating the risk of RIBI.

A receiver operating characteristic (ROC) curve for the nomogram demonstrated an area under the curve (AUC) of 0.93.

## Discussion

Radiation induced bone injury (RIBI) has been reported as the radiotherapy toxicity in a number of studies [2,3,5,6,10-14]. The incidence of RIBI in patients treated with lung SBRT has been variably reported as ranging from 0% to more than 50% [4,5,15]. The variability may be due to differences in treatment technique and dose-fractionation, reported outcomes, selection criteria, follow up procedures, whether or not available radiography was reviewed again for rib fractures, and the process of analysis. For example, Pettersson et al. [3] analyzed the planning information of 33 patients treated with 45 Gy in 3 fractions. With a median follow up of 29 months, 13 fractured ribs were identified in 7 patients. They estimated that delivering 27.3 Gy to 2 cm^3^ (D_2_) of ribs was associated with a 50% risk of fracture. In this study ribs receiving less than 21 Gy were excluded. In our study the value of D_0.5_ had the maximum likelihood (MLL) value; however we included all the ribs in our analysis. To evaluate the impact of including the ribs receiving low dose RT, we repeated the MLL curves excluding the ribs receiving < 25 Gy in a stepwise process however, the value of D_0.5_ remained the significant MLL cut point. In our cohort, a 50% risk of rib fracture was associated with a D_0.5_ of 60 Gy.

This was consistent with data from Stephans et al. [12] who found that in 45 patients treated with 60 Gy in 3 fractions there was no chest wall toxicity observed with a minimum absolute chest wall point dose of less than 67.5 Gy.

Similar findings have been reported by Onishi et al. in abstract form [16]. In this study with a median follow up of 33 months, RIBI was observed in 41 (23.2%) patients. BED calculation with α/β ratio of 3 was used for dose comparison. There was no rib fracture observed in patients in whom the maximum point dose to the chest wall was less than 218 BED (approximately equal to 40 Gy in 3 fractions) but rib fracture was considered “inevitable” when the BED was more than 250. Although there is controversy surrounding the use of the LQ model in SBRT [17,18] this data supports a dose–response relationship for rib fracture. Explanations for Pettersson et al. [3] having a 50% risk of RIBI with lower SBRT doses could include confounding clinical variables, and the small sample size. The relationship between delivered dose to the ribs and the risk of fracture has also been studied by Chollet et al. [19] who found no rib fractures in 15 patients treated with 50 Gy in 5 fractions within the median FU of 13 months. Although a lower maximum point dose to the chest wall might be related to the lack of event in these patients, the potential risk for chest wall toxicity should be weighed carefully against the potential benefit of higher SBRT dose in terms of tumor control probability [20].

In our study, D_0.5_ and other dosimetric parameters were all correlated with the risk of developing RIBI but inclusion of clinical variables, notably age and gender, improved the predictive model. We have created a nomogram based on these 3 dosimetric and clinical parameters. As an illustration (Figure 6), a 75 year old woman who received a planned dose of 60 Gy to 0.5 cc of a rib has an estimated 65% risk of RIBI within the first two years of follow up. A man of the same age and with the same D0.5 would in contrast have about a 15% risk of RIBI.

Strengths of our study include the long median follow up time (25 months) and careful radiologic review. As RIBI is a late toxicity, to accurately assess event rate, it is important to follow these patients closely, not only with clinical exam but because many rib fractures are asymptomatic, also with serial CT scans. Initially, radiology reports did not always identify the presence of a new rib fracture. Fifteen fractured ribs were not reported and overall there was an average of 5 months latency in reporting fractured ribs. This highlights the importance of spreading knowledge in the radiology community about the pattern of late toxicity that can be seen with SBRT. Furthermore, to minimize potential sources of error, our group of patients was selected to be as homogenous as possible - all had more than 6 months follow up and all were treated with 54 or 60 Gy in 3 fractions. Additional strengths are: prospective data collection as part of REB-approved institutional protocol [6], exclusion of patients with other causes of rib fractures such as bone metastases or trauma, standard contouring of ribs and planning, evaluating multiple different DVH values, and including clinical and dosimetric factors.

Symptomatic chest wall toxicity has been observed in patients with lung cancer treated with stereotactic radiotherapy [21,22]. Dunlap et al. [4], reported chest wall pain in 20 and rib fracture in 5 out of 60 lung SBRT patients treated with various dose fractionation schedules. Their analysis only included those patients with tumors located within 2.5 cm of chest wall or those whose maximum point dose to the chest wall exceeded 20 Gy. With median follow up of 11 months, they reported 30% risk of chest wall pain or rib fracture if 35 cm^3^ of the chest wall received more than 30 Gy. The fact that the ribs were not evaluated separately and the patients received several dose fractionation schedules makes it difficult to compare their results to the current study. In our cohort, 14 patients with rib fracture had chest wall pain (in comparison to 7 patients without rib fracture). The majority of these cases had grade 1 or 2 chest wall pain however; there were 3 cases of grade 3 chest wall pain in the group of patients with rib fracture (Figure 3). Moreover ribs received statistically significant higher dose in patients with chest wall pain in comparison to ones without chest wall pain (Table 4). This justifies an attempt to reduce the dose to the ribs if and when possible. The dose constraints identified are most useful in situations where the tumor is sufficiently far away from ribs that planning and optimization efforts to reduce dose can be useful without decreasing tumor dose or increasing lung dose. Attention to radiation planning technique in order to limit hot spots/D_0.5_ in the chest wall and adjacent ribs, without compromising PTV coverage may be beneficial. Currently, it is our institutional policy to contour any ribs adjacent to the PTV and attempt to spare them without compromising PTV coverage. Advanced RT techniques such as VMAT and IMRT might also help with this. Our group has chosen to use the 48 Gy in 4 fraction schedule for tumors less than 3 cm that are immediately adjacent to the chest wall as we have not yet observed a high rate of fracture in this group while tumor control remains excellent.

Our study had a number of limitations. First, the study set was limited to patients with three fractions; it is unclear if the model derived will have similar correlations with RIBI in patients treated with different dose fractionations. Second, due to the small sample size and limited events, it was not possible to divide the data into training and testing sets to allow internal model validation. Therefore, our nomogram model requires subsequent validation on another dataset. Nevertheless, it may help to improve the general understanding of RIBI risk and to emphasize the need for clear discussion with potentially high-risk patient groups who are treated with SBRT. Third, the clinical factors explored in the current study were limited by data availability. There are other clinical factors that could potentially play a role in RIBI, such as cough, corticosteroid use, and presence of osteoporosis that should be explored in future investigations. In addition, the dosimetric study was based on rigid rather than deformable registration and on planned rather than received dose. Several factors may play a role in determining the actual dose received by the ribs such as variation in daily positioning and breathing motion. Assessment of cone beam set up images in combination with deformable image registration and dose accumulation may help to identify the impact of these factors and suggest further risk reduction strategies.

## Conclusions

Radiation oncologists, diagnostic radiologists and other specialists who see patients post SBRT, as well as patients themselves should be aware of and informed about the late toxicities related to lung SBRT, including rib fracture. Risk factors for RIBI include increasing age, female gender, and high RT dose to 0.5 cc of nearby ribs. A nomogram incorporating these factors may be useful in estimating individual patient risk, though internal and external validation of this model is needed.

## Competing interests

This study was supported in part by Elekta Oncology Systems.

## Authors’ contributions

MT; has made substantial contributions to conception, design, acquisition of data, analysis, interpretation of data, drafting and revising of the manuscript. AH; has made substantial contributions to conception, design, interpretation of data, drafting and revising of the manuscript. PL; has made substantial contributions to conception, and acquisition of data. MD; has made substantial contributions to conception, design, interpretation of data, drafting and revising of the manuscript. SF; participated in the design of the study and performed the statistical analysis. TP; has made substantial contributions to drafting the manuscript. DJ; has made substantial contributions to interpretation of data, and drafting the manuscript. LD; has made substantial contributions to conception, design, interpretation of data, drafting and revising of the manuscript. AB; has made substantial contributions to conception, design, interpretation of data, drafting and revising of the manuscript. All authors read and approved the final manuscript.
